# Low dietary diversity and associated factors among lactating mothers in Angecha districts, Southern Ethiopia: community based cross-sectional study

**DOI:** 10.1186/s13104-018-4001-6

**Published:** 2018-12-14

**Authors:** Moges Muluneh Boke, Alehegn Bishaw Geremew

**Affiliations:** Department of Reproductive Health, Institute of Public Health, College of Medicine and Health Science, University of Gondar, 196, Gondar, Ethiopia

**Keywords:** Low dietary diversity, Lactating mothers, Ethiopia

## Abstract

**Objective:**

The objective of the study was to assess prevalence low dietary diversity and associated factors among lactating mother in Angecha district, Southern Ethiopia.

**Results:**

The magnitude of low dietary diversity was 52.2%, 95% confidence interval (47.4, 57.27). From multivariable logistic analysis mothers were illiterate had 2.5 times more likely to have low dietary diversity than had formal education. Mothers living in the rural area were 3.1 times more likely to have low dietary diversity as compared with living urban area and women who have Household food insecure was 3.4 times more likely to have low dietary diversity as compared to a counterpart. Therefore, health provider could provide nutritional education focusing illiterate and rural women.

**Electronic supplementary material:**

The online version of this article (10.1186/s13104-018-4001-6) contains supplementary material, which is available to authorized users.

## Introduction

Individual dietary diversity is a qualitative measure of food consumption and it reflects household access to a variety of foods [1], it also indicates the level of nutrient adequacy, in many countries evidence shown that increasing individual dietary diversity score is related with improving nutrient adequacy of the diet [2]. Individual dietary diversity could be assessed by using the dietary score that means the sum the number of food groups consumed over a reference period [3].

Promotion of diverse diets is one of several approaches to improving micronutrient nutrition for women as well as lactating women [4]. Consumption of verity of a food group provides different essential nutrients to body normal growth as well as prevention of any disease, while low dietary diversified leads to malnutrition [5].

In most developing countries micronutrient malnutrition among lactating mothers is a huge problem due to a different reason, the first is due to physiological change like pregnancy and lactation, during lactating mothers lose protein, other nutrients through breast milk due to this demand need of nutrients increases [6, 7]. The other reason for maternal micronutrients malnutrition is inadequate intake. The feeding practice of developing country mothers is non-diversified like only starch-based foods and no animal products, vegetable and fruits [8].

In Ethiopia evidence of dietary pattern among lactating mothers is scarce, and in some study evidence show that starch staples based diet like rice, sorghum, barley, wheat are the most common diet and it is consumed by 9 of every 10 households per week [9], additional to this in another study carried out in Northern part of Ethiopia observed poor dietary diversity among lactating mother is prevalent, and more than half of study participants have low dietary diversity [10], Average monthly income, home garden, source of drinking water, maternal educational status, food insecurity and socio-economic status reported as risk factors in different previous study [10, 11].

To alleviate nutrition-related health problem, Ethiopian government implemented different intervention programs such as linking with maternal continuum care, micro-nutrients supplementation to pregnant and lactating mothers, implementing packages of Health Extension Program (HEP) and nutrition education like dietary diversification, but still the prevalence and burden of malnutrition is prominent problem [12, 13]. Therefore, the aim of this study was to assess the magnitude of low dietary diversity and associated factors among lactating mothers in Angecha, Southern Ethiopia.

## Main text

### Methods

#### Study design and period

A community-based cross sectional study design was conducted from March to April 2017, among lactating mothers in Angecha district, Southern Ethiopia.

#### Study setting

The study was conducted in Angecha district, Southern Ethiopia. The administrative center of Angecha district is Angecha town, which is located 255 km to the south of Addis Ababa, the capital city of Ethiopia. According to the district health office report, a total of 112,450 individuals were living in 5510 households. An estimated to 4273 lactating mother present in the district during the data collection period. The major agricultural products of the district are wheat, barley, teff, sorghum, maize, pea, and beans and some vegetables like cabbage, potato, carrots, inset and lettuce (Unpublished report from the district agricultural office). The district has 20 health posts, 5 health centers and 4 private clinics (South nation, nationality people regional health bureau report, 2016). Lactating mothers who have a child age less than 2 years was the study population and lactating mothers who lived in the study area at least for 6 months were included in the study.

#### Sample size determination

A single population proportion formula was used with the assumption of 95% confidence level, 4.5% margins of error, and the prevalence of low dietary diversity among lactating mothers was 56.4% [10] and 10% non-response rate, finally, the estimated sample size for this study was 426.

#### Sampling technique

A simple random sampling technique was employed to select study participants. The sampling frame was prepared by reviewing health post family folder and records from all 20 kebeles of the district. A total of 2825 lactating women were identified through reviewing health post family folder and record form, when more than one lactating women found during preparing sampling frame one women selected using lottery methods. Finally, 426 lactating mothers were selected by using computer-generated random number method.

#### Measurements

*Knowledge on nutrition* The level of knowledge on nutrition was measured using scores obtained on the nutrition knowledge questions: less than 33% indicated the women had poor knowledge, 33–66% indicated fair knowledge while above 66% indicated good knowledge [14].

Individual/women dietary diversity score was calculated by summing the number of food groups consumed by the mother over the 24-h recall period. Nine food groups were proposed for the Women Dietary Diversity Score (WDDS). The nine food groups used to calculate WDDS are Starchy staples (cereals and white tubers), dark green leafy vegetables, other vitamins A rich fruits and vegetables, other fruits and vegetables, organ meat, meat and fish, eggs, legumes, nuts and seeds, milk and milk products.

*Low dietary diversity* Lactating mother the sum of food groups eaten in the previous 24 h less than the mean value.

#### Data collection instrument and procedures

A structured questionnaire was formulated from different literature [10, 15–17]. Household Food Insecurity Access questionnaire, was adapted from a validated Household Food Insecurity Access questionnaire developed by the Food and Nutrition Technical Assistance [1, 4]. The questionnaire includes socio-demographic and economic characteristics, reproductive history, household food security, knowledge of nutrition, dietary diversity intake. Four clinical nurses data collectors and two B.Sc nurse supervisors who are fluent in speaking and writing of local language were recruited. The data were collected through face to face interview administered. The mother’s dietary diversity status was measured by a recall of all foods consumed by each woman during the previous 24 h.

#### Data quality management

To assure the quality of the data, the questionnaire was prepared in the English version and translated into the local language of the respondents and again translated back to English. The questionnaire was pretested among 5% of the study sample outside the study area and necessary modification was made accordingly. The data collectors and supervisors were given 2 days training on data collection technique and procedure. Supervision was done by supervisors to checked completeness, and consistency of the collected data throughout the data collection period, and the overall supervision of data collection processes were done by the principal investigators.

#### Data processing and analysis

Each questionnaire was checked manually, coded and entered into EPI info version 3.54 and imported to statistical package of social science (SPSS) Version 20.0 for analysis. The descriptive results expressed using summary statistics such as mean, standard deviation, frequency, and percentage. A binary logistic regression model was fitted to determine the effect of various factors with low dietary diversity. Variables with a *p* value less than 0.2 in bivariate analysis were entered into multivariable logistic regressions to determine factors independently associated with low dietary diversity. The odds ratio with a 95% confidence interval was used to determine factors independently associated with low dietary diversity.

### Results

#### Socio-demographic characteristics of the study participants

Four hundred and ten lactating mothers participated in a response rate of 96.2%. The mean age of study participants was 28.1 ± SD 5.24 years. From the participant’s majority were Kembata ethnic group (81%), rural resident (80.5%), and married (96.3%). More than half (56.6%) of study participants were illiterate and nearly three-fourths (74.1%) of women occupation were a housewife and 365 (89.0%) live in male-headed households. The mean family size and a number of under-five children were 5.01 (± 1.6 SD), and 1.1 (± 0.33 SD), respectively. Based on the Household Food Insecurity Access Scale (HFIAS) measurement, 57 (13.9%) of study participants households food insecure. Out of the participants, 20% of family wealth index were very poor (Table 1). Among the respondents, 269 (65.6%) were experiencing pregnancy for ≤ 3 times and three hundred and twenty-five (79.2%) received antenatal care (ANC) at least once during their last pregnancy, but only 43.3% had ANC visits of greater than or equal to 4 times. Almost all, 401 (97.3%) of the study subjects did not avoid any food within the lactation period because of cultural/traditional reasons, whereas, only 21 (5.6%) of them were eating additional foods during their lactation period. Out of the total participants, 49.1% had good nutritional knowledge, while 8.1% of participants had poor knowledge (see Additional file 1).

**Table 1:** Socio-Demographic and household characteristics of lactating mothers in Angecha district, Southern Ethiopia, 2017 (n = 410)

Variables	Frequency	Percentage (%)
Age (years)		
17–25	180	43.9
26–35	198	48.3
36–49	32	7.8
Marital status		
Married	395	96.3
Divorced	7	1.7
Other*	8	2.0
Residence		
Rural	330	80.5
Urban	80	19.5
Ethnicity		
Kembata	336	81
Hadiya	54	13.2
Amhara	17	4.1
Religion		
Protestant	285	69.5
Orthodox	61	14.9
Adventist	42	10.2
Catholic	21	5.1
Adventist	42	10.2
Other**	1	0.2
Educational status of mother		
Illiterate	232	56.6
Formal education	178	43.4
Mother occupation		
Farmer	49	12.0
Housewife	304	74.1
Gov employment	28	7.6
Other***	31	6.3
Husband educational status		
Illiterate	178	43.4.1
Formal education	193	52.0
Husband occupation		
Farmer	224	56.6
Gov employment	71	17.3
Private work	60	14.6
Other****	15	3.6
Head of household		
Mother	41	10.0
Husband	365	89.0
Other*****	4	1.0
Family member size		
1–4	165	40.2
Greater than 4	245	59.8
Number under 5-year child		
One	359	95.1
Greater than 1	51	12.4
Household food insecurity level		
Food secure	353	86.1
Food insecure	57	13.9
Wealth index		
Very poor	82	20.0
Poor	82	20.0
Medium	88	21.5
Rich	76	18.5
Very rich	82	20.0
Source of water		
Pipe	321	78.3
Protected	84	20.5
Unprotected	5	2.5
Type of toilet facility		
Traditional	395	96.3
Improved	15	3.7

* Include never married, divorced

** Include Muslim and no religion

*** Include daily labor, private work

**** Daily labor

***** Her/his father or mother and grandparents

#### Dietary diversity characteristics

**Fig. 1:**
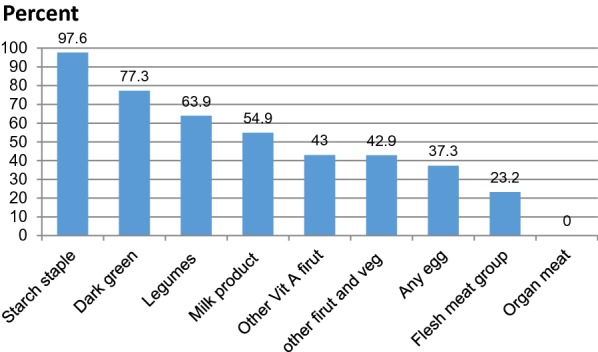
Percentage of food groups consumed by respondents in the past 24 h, among lactating mothers live in Angacha district southern Ethiopia 2017

The dietary diversity score was ranged from 1 to 8 and mean dietary diversity score among the lactating mothers was 4.5 (± 1.58 SD). Among the total lactating mothers, 52.2%, 95% CI (47.4, 57) had low dietary diversity in the previous 24 h. Starchy staples including cereals and white roots and tubers were the most frequently consumed food group (97.6%) 24 h proceeding data collection time, followed by fruits and vegetables (77.3%). The least consumed food group was flesh meat (23.2%) and organ meat (liver, kidney, and heart) was not consumed by lactating mothers (Fig. 1).

#### Factors associated with low dietary diversity

In the multivariable logistic regression analysis, the covariates: educational status of mother, residence, and household food insecurity status were statistically significant at 5% and were found to be factors associated with low dietary diversity among lactating mothers.

Educational status of lactating mothers participated in this study, those mothers who were illiterate were 2.3 times more likely to have low dietary diversity than those mothers who had formal education (AOR = 2.3, 95% CI 1.34–3.99), and mothers living in rural area were 3.1 times more likely to have low dietary diversity as compared with living urban (AOR = 3.1, 95% CI 1.78–4.63). Lactating mothers from food insecure households were 3.4 times more likely to have low dietary diversity as compared with from food secure households (AOR = 3.4, 95% CI 1.09–10.8) (Table 2).

**Table 2:** Bivariate and multivariable logistic analysis showed factors associated with low dietary diversity among lactating women in Angacha district, 2017

Associated factors	Category	Low dietary diversity		COR (95% CI)	AOR (95% CI)	P-value
		Yes	No			
Age (years)	17–25	109	71	3.9 (1.72, 8.97)	4.2 (0.49, 11.52)	0.041
	26–35	96	102	2.4 (1.06, 5.48)	2.1 (1.14, 6.83)	0.29
	36–49	9	23	1	1	
Residence	Rural	192	138	3.7 (2.14, 6.28)	3.1 (1.78, 4.63)	0.00*
	Urban	22	58	1	1	
ANC category	No ANC	48	49	1.2 (0.74, 2.09)	0.9 (0.50, 1.56)	0.66
	< 4 time	106	71	1.9 (1.20, 2.97)	1.5 (.94, 2.53)	0.88
	≥ 4 time	60	76	1	1	
Educational status of mother	Illiterate	148	93	0.4 (0.27, 0.60)	2.5 (1.62, 3.88)	0.00*
	Formal education	66	103	1	1	
HFIAS category	Food secure	169	184	1	1	
	Food insecure	45	12	4.1 (2.09, 8.98)	3.4 (1.09, 10.8)	0.036*
Wealth index	Very poor	28	54	0.4 (0.21, 0.73)	0.6 (2.85, 1.27)	0.18
	Poor	47	35	1.0 (0.54, 1.86)	1.1 (0.56, 2.18)	0.77
	Medium	56	32	1.3 (0.70, 2.4)	1.3 (0.69, 2.54)	0.40
	Rich	36	40	0.7 (0.36, 1.2)	0.7 (0.35, 1.35)	0.28
	Very rich	47	35	1	1	

*AOR* Adjusted Odd Ratio, *CI* confidence interval, *COR* Crude Odd Ratio, *HFIAS* Household Food Insecurity Access

* Significant at 0.05

### Discussion

Low dietary status among lactating mother is attribute for maternal and child under nutrition, so that this study has assessed the prevalence of low dietary diversity and its associated factors among lactating mothers living Angacha districts southern, Ethiopia. The mean DDS among the participants was 4.5, this finding is lower than that of studies conducted in Jimma, Ethiopia, and Vietnam [11, 19], this difference could be but higher than study in Northern Ethiopia, and rural Bangladesh [10, 20]. This might be study in northern Ethiopia had high food insecurity status.

Our finding revealed that 52.2% (95% CI (47.4, 57) of women had a low dietary intake. This finding was much higher than studies finding reported in Jimma Ethiopia, Bangladesh, and Vietnam [11, 18]. This might be explained by the difference in study period which could result in food security status change. The study in Jimma Ethiopia, only 6.5% of the study participants were food insecure. While in the present study, 13.9% were food insecure. There is also a similar difference in antenatal care follow up visits between study participants of both studies which could be another evidence for the showed discrepancy, mothers when visiting health facility for antenatal care, they receive different nutritional education, so it might help to improve their dietary diversity habit. However, our finding was lower than from study conducted in northern Ethiopia [10]. The possible explanation for this difference could be high household food insecurity in the previous study.

In this study educational status, household food insecurity and residence of mothers were factors associated with low dietary diversity. Mothers who had illiterate were 2.5 times more likely to had low dietary diversity than those mothers who had formal education. This finding is supported by other evidence [11]. This might be Education is important to improve the knowledge of dietary diversification and long-term behavioral change. A mother from food-insecure households was 3.4 times more likely to have low dietary diversity intake when compared with those mothers from food secure households. This result also in similar with studies conducted in Bangladesh, Vietnam, and Ethiopia [11, 19]. A mother living in the rural area were 3.1 times more likely to had low dietary diversity as compared with living urban area, this in line with a study conducted in South African [20]. This might be due to low cognitive accessibility of the rural residence.

### Conclusions

Our study identified 52.2% of the lactating mother had low dietary status in the study area. Educational status (illustrate), households with food-insecure status, and residence (rural) were statistically associated with low dietary diversity.

Therefore, Nutritional counseling focusing on illiterate women will be considered during health service utilization and community based educational campaign through health extension worker for lactating women might solve the problem.

### Limitations of the study

The major limitation of this study was seasonal variation which might affect food availability in the household this intern cause low dietary diversity status but no activities were implemented to addressee on seasonal variation regards. The study was relying on 24-h dietary recall which does not show the usual dietary practice of household members and affected by religious festivals.

## Additional file

**Additional file 1: Table S1.** Reproductive health-related factors of lactating mother in Angacha district, 2017.

## Abbreviations

ANC: Ante Natal Care
AOR: Adjusted Odds Ratio
COR: Crude Odds Ratio
CI: confidence interval
DD: dietary diversity
HEP: Health Extension Program
HFIAS: Household Food Insecurity Access Scale
SPSS: statistical package of social science
