# Supplementary material for: Low dietary diversity and associated factors among lactating mothers in Angecha districts, Southern Ethiopia: community based cross-sectional study
Source: BMC Res Notes. 2018 Dec 14;11:892. doi: 10.1186/s13104-018-4001-6 (PMC6295037; doi:10.1186/s13104-018-4001-6)
Supplement: Supplementary file 1 — Additional file 1: Table S1. Reproductive health-related factors of lactating mother in Angacha district, 2017. [file 13104_2018_4001_MOESM1_ESM.docx]

Table S1: Reproductive health-related factors of lactating mother in Angacha district, 2017

| **Variables** | **Category** | **Frequency** | **Percentage (**%) |
| --- | --- | --- | --- |
| **ANC visits** | No ANC | 85 | 20.7% |
|  | < 4 | 184 | 44.9% |
|  | ≥ 4 | 141 | 34.4% |
| **Cultural avoided food** | Yes | 9 | 2.2% |
|  | No | 401 | 97.8% |
| **Women meals frequency per day** | <4 | 389 | 94.9% |
|  | ≥4 | 21 | 5.1% |
| **Mother nutritional knowledge** | Poor | 33 | 8.1% |
|  | Fair | 172 | 42.1% |
|  | Good | 204 | 49.9% |
